# Supplementary material for: Morphological and functional cardiac alterations in children with congenital Zika syndrome and severe neurological deficits
Source: PLoS Negl Trop Dis. 2023 Nov 29;17(11):e0011762. doi: 10.1371/journal.pntd.0011762 (PMC10712894; doi:10.1371/journal.pntd.0011762)
Supplement: Attachment — Submitted filename: Response to reviewers.docx [file pntd.0011762.s005.docx]

Answer to reviewers

Dear Editor,

We sincerely appreciate your dedication to reviewing the article. The responses to each topic are included below.

Reviewer 1: I appreciate the considerations; they were important for improving the article.

Methods

1) The determination/consideration of the study's sample size or power seems to have not been delineated.

Answer: I described the rationale behind the sample size in lines 128 through 132. “The sample was convenience-based, including all patients with CZS under follow-up at the research center in 2021. There was limited adherence in the control group since data collection occurred during the COVID-19 pandemic. Considering that the values would be adjusted by the Z score and reference values are well defined in the literature, we chose to maintain a 2:1 ratio.”

The parents did not adhere, the children were not vaccinated, and the fear of COVID-19 was still very high. This information was also included in the discussion section, where we addressed the study's limitations. Lines 459 through 462: “Additionally, the COVID-19 pandemic limited the ability of patients residing in other states to access the IPESQ research center and made it difficult for parents to allow the children in the control group to attend medical clinics.”

2) The current manuscript only used bivariate analysis to access the epidemiological associations, and extra multivariate analyses adjusting for confounders are needed.

Answer: Thank you for the suggestion. We have added the following paragraph to the results – lines 263 through 270:

“We employed a linear regression model to analyze the relationships among confounding variables. Having CZS, after adjusting for sex, age, weight, and height, negatively affected the left atrial volume index (p<0.01), right atrial volume index (p<0.01), left ventricular end-diastolic diameter (p<0.01), basal right ventricular diameter (p<0.01), S' velocity of the right ventricular lateral wall (p=0.09), tricuspid annular plane systolic excursion (p<0.01), and mitral annular plane systolic excursion (p<0.01) and had no effect on the ejection fraction (P = 0.354).

Results

1) The footnotes in Tables can be strengthened.

I organized the notes, corrected some abbreviations, and arranged them alphabetically.

2) When prevalence estimate of a health outcome is presented, the author can consider providing its 95% confidence interval to indicate uncertainty,

Answer: I would have liked to, but since the numerical outcomes were presented as the median and range, the 95% confidence interval for this parameter was not routinely provided.

3) Some prevalence estimates may be summarized in new tables to improve readability.

Answer: We added a paragraph in the results section showing the multivariate analysis – lines 263 through 270: “We employed a linear regression model to analyze the relationships among confounding variables. Having CZS, after adjusting for sex, age, weight, and height, negatively affected the left atrial volume index (p<0.01), right atrial volume index (p<0.01), left ventricular end-diastolic diameter (p<0.01), basal right ventricular diameter (p<0.01), S' velocity of the right ventricular lateral wall (p=0.09), tricuspid annular plane systolic excursion (p<0.01), and mitral annular plane systolic excursion (p<0.01) and had no effect on the ejection fraction (P = 0.354).”

Conclusion:

1) The public health implications for the population at large seem to have not been mentioned in the manuscript. Rather, the current conclusion paragraph seems to focus solely on the next steps for the study population.

Answer: Thank you for the suggestion. We improved the conclusion by adding more information, in lines 463 to 471: “In conclusion, although congenital Zika syndrome is a rare condition, its impact is profound, causing serious neurological damage and affecting other organs. This study reveals a concerning reduction in the size and functional changes of the heart. It is crucial to conduct further research to better understand how the heart is affected, whether it is due to direct viral action or nutritional deficits. Early identification of cardiac anomalies underscores the importance of conducting regular cardiac assessments and providing continuous follow-up for this group of patients, who often face vulnerabilities and heavily rely on access to public healthcare services.”

Reviewer 2: I appreciate the considerations; they were important for improving the article.

Methods

1) The population description for the CZS group is well described, however, there are very few details of the healthy group, I would suggest that they do a better description of the population and specify if they applied any selection criteria.

Answer: I described more details of the control group – lines 123 through 125: “The control group comprised 25 healthy children of the same age and gender range from public schools in Campina Grande, Paraíba, and the invitation to participate was extended during school meetings”

2) I believe that the 2:1 ratio of cases and controls should be reviewed. If there is any reason why they made the decision of this relationship, it would be interesting if they describe it in the discussion, since for the results achieved, I do not believe that the sample size is adequate. I understand the characteristics of the study population, but it would be nice to increase the controls. If there is any support for that decision, ignore this recommendation.

Answer: I described the rationale behind the sample size in lines 128 through 132. “The sample was convenience-based, including all patients with CZS under follow-up at the research center in 2021. There was limited adherence in the control group since data collection occurred during the COVID-19 pandemic. Considering that the values would be adjusted by the Z score and reference values are well defined in the literature, we chose to maintain a 2:1 ratio.”

The parents did not adhere, the children were not vaccinated, and the fear of COVID-19 was still very high. This information was also included in the discussion section, where we addressed the study's limitations. – lines 459 through 462: “Additionally, the COVID-19 pandemic limited the ability of patients residing in other states to access the IPESQ research center and made it difficult for parents to allow the children in the control group to attend medical clinics.”

Conclusion:

1) They could improve the recommendation of the line 424 – 426

Answer: Thank you for the suggestion. We improved the conclusion by adding more information in lines 463 to 471: “In conclusion, although congenital Zika syndrome is a rare condition, its impact is profound, causing serious neurological damage and affecting other organs. This study reveals a concerning reduction in the size and functional changes of the heart. It is crucial to conduct further research to better understand how the heart is affected, whether it is due to direct viral action or nutritional deficits. Early identification of cardiac anomalies underscores the importance of conducting regular cardiac assessments and providing continuous follow-up for this group of patients, who often face vulnerabilities and heavily rely on access to public healthcare services.”

2) I have a recommendation in this regard, in line 279 - 283, it mentions one of the strengths of this study that presents original and novel results, however it would be important to mention that in vitro studies have been carried out, you can review the following article, it may help you as an important precedent for its results.

Answer: I would like to thank you for the suggestion to include this in the article; it enriched the study. I have included a paragraph discussing this matter, lines 319 through 327: “Rossi and colleagues conducted an in vitro study that characterized ZIKV infection of human fetal cardiac mesenchymal stromal cells (fcMSCs), which play important roles in both normal and pathophysiological cardiac development and function. Their findings indicate that ZIKV predominantly enters cells using the TAM-family protein tyrosine kinase receptor AXL, affecting the fcMSC differentiation process and cell death. These observations strongly suggest that heart tissue may represent another susceptible target of ZIKV infection; however, the potential cardiac-related effects of ZIKV infection have not been well characterized (23).”

Editorial and Data Presentation Modifications

1) The sentence of line 121 I think is unnecessary. The specification that they were born to mothers who were infected during pregnancy could be added to lines 116 and 117.

Answer: We have adjusted the paragraph as suggested – lines 116 through 119: “Between 2021 and 2022, we recruited 52 children whose mothers were infected with ZIKV during pregnancy who were diagnosed with CZS and receiving follow-up care at the Joaquim Amorim Neto Research Institute (IPESQ) in Campina Grande, Paraíba, Brazil for our cross-sectional study.”

Reviewer 3: I appreciate the considerations; they were important for improving the article.

Methods

1) The methods section needs to be rewritten as there are several repetitions.

Answer: We organize the text to avoid repetition.

2) Lines 127 to 129 is an irrelevant information from the perspective of the manuscript.

Answer: The graduate program where I am pursuing my Ph.D requires the inclusion of this information. I would appreciate it if you could consider retaining it.

Results

1) In the opinion of this reviewer, the work is clinically relevant and sheds light on the pathologies associated with CZS. However, this is purely clinical work that was done in a single center. While the clinical data is unequivocal and thoroughly done, the authors do not offer any mechanistic explanations for the same. Could the authors attempt to provide such explanations?

Answer 1: The research was conducted at a single center; however, IPESQ has become a reference center for the disease, with multiprofessional treatment, serving children from several states of Brazil, as well as children from other countries. In our sample, there is a child who was born in Africa, and the mother chose to come to live in Campina Grande to receive better health treatment.

Answer 2: I greatly appreciate the request for this explanation; it has enriched the work. We are not certain about the cause of this, but we believe it is related to the overall growth deficit or there is a possibility that the heart may be affected in the same way as skeletal muscle is. We have added the following paragraphs to the discussion:

Lines 328 through 338: “Another study examined ZIKV replication in human skeletal muscle myoblasts, revealing that it impairs muscle fiber differentiation. In mouse models, it was demonstrated that ZIKV targets muscle tissue both during embryonic development and after birth. This muscle infection resulted in necrotic lesions, inflammation, fiber atrophy, and reduced expression of myogenic factors, indicating that skeletal muscle is a site of viral amplification and injury that impairs muscle development. Additionally, the study suggested that ZIKV infection during myoblast differentiation could reduce the number and area of newly formed muscle fibers, potentially causing damage during fetal and neonatal muscle development (35). The heart is potentially affected in the same way as the skeletal muscle.

Lines 421 through 431: “Instances of critical illness in infants and children often imply a hypermetabolic state. Children with cerebral palsy commonly manifest nutritional and growth disorders, occasionally requiring gastrostomy tube feeding due to severe malnutrition, dysphagia, feeding difficulties, and the risk of broncho-aspiration (33). Substantial nutritional deficits can impede musculature development, potentially impacting cardiac musculature. Aguiar et al. observed that between the ages of 25 and 36 months, over 50% of CZS children fell below weight and height thresholds, with growth restriction being evident since gestation (34). The growth deficit in CZS likely results from a combination of factors, and our findings suggest that the heart may also be affected by this growth restriction.”

Once again, I thank all the reviewers for their suggestions; I am confident that the article has been greatly improved with the incorporation of the modifications. I hope I have addressed the questions raised.

Kind regards,

Imara Queiroz
